# Microbiota Modulation of Radiosensitiveness and Toxicity in Gastrointestinal Cancers: What Radiation Oncologists Need to Know—A Review on Behalf of the Italian Association of Radiobiology (AIRB)

**DOI:** 10.3390/cimb47040265

**Published:** 2025-04-09

**Authors:** Marco Lorenzo Bonù, Andrea Georgopulos, Marco Ramera, Jacopo Andreuccetti, Andrea Emanuele Guerini, Anna Maria Bozzola, Vittorio Morelli, Jacopo Balduzzi, Mirsada Katica, Mariateresa Cefaratti, Lorenzo Granello, Luca Triggiani, Michela Buglione, Stefano Maria Magrini, Francesco Marampon, Michele Mondini, Silvana Parisi, Giorgia Timon, Luisa Bellu, Maria Rescigno, Stefano Arcangeli, Marta Scorsetti

**Affiliations:** 1 Department of Radiation Oncology, Istituto del Radio O. Alberti, Spedali Civili Hospital, 25123 Brescia, Italy; 2 Department of Radiation Oncology, Spedali Civili Hospital and Brescia University, 25123 Brescia, Italy; a.georgopulos@unibs.it (A.G.); a.e.guerini@gmail.com (A.E.G.); vittoriomorelli.dr@gmail.com (V.M.); j.balduzzi@unibs.it (J.B.); m.katica@unibs.it (M.K.); m.cefaratti@unibs.it (M.C.); l.granello@unibs.it (L.G.); luca.triggiani@unibs.it (L.T.); michela.buglione@unibs.it (M.B.); stefano.magrini@unibs.it (S.M.M.); 3 Department of Surgery, Spedali Civili Hospital and Brescia University, 25123 Brescia, Italy; 4 Department of Surgery, Spedali Civili Hospital, 25123 Brescia, Italy; jacopo.andreuccetti@gmail.com; 5 Department of Pathology, Spedali Civili Hospital, 25123 Brescia, Italy; anna.bozzola@gmail.com; 6 Department of Radiological Oncological and Pathological Sciences, Sapienza University of Rome, 00185 Rome, Italy; francesco.marampon@uniroma1.it; 7 Department of Radiation Oncology, Gustave Roussy Cancer Campus, 94800 Villejuif, France; michele.mondini@gustaveroussy.fr; 8 Radiation Oncology Unit, Department of Biomedical, Dental Science and Morpological and Functional Images, University of Messina, 98122 Messina, Italy; dott.silvanaparisi@gmail.com; 9 IRCCS Ospedale Policlinico San Martino, 16132 Genoa, Italy; giorgia.timon@hsanmartino.it; 10 Department of Radiotherapy and Radiosurgery, IRCCS Humanitas Research Hospital, 20089 Rozzano, Italy; luisa.bellu@cancercenter.humanitas.it (L.B.); marta.scorsetti@hunimed.eu (M.S.); 11 IRCCS Humanitas Research Hospital, 20089 Rozzano, Italy; maria.rescigno@unimed.eu; 12 Department of Biomedical Sciences, Humanitas University, 20072 Milan, Italy; 13 Radiation Oncology Unit, Fondazione IRCCS San Gerardo Dei Tintori, 20900 Monza, Italy; stefano.arcangeli@unimib.it; 14 Medicine and Surgery Department, University of Milan Bicocca, 20126 Milano, Italy

**Keywords:** radiotherapy, microbiota, gut microbiota, modulation of toxicity, modulation of efficacy

## Abstract

The impact of the microbiota on radiation (RT)-induced toxicity and cancer response to radiotherapy is an emerging area of interest. In this review, we summarize the available preclinical and clinical evidence concerning microbiota modulation of RT toxicity and efficacy in the main gastrointestinal (GI) districts. A huge amount of data supports the clinical application of microbiota modulation, particularly through prebiotics and probiotics, to prevent or mitigate radiotherapy-induced toxicity in rectal cancer. Preclinical and clinical studies also support the observation of microbiota modulation to impact the toxicity and efficacy of treatment in esophageal cancer, hepatocellular carcinoma (HCC), and anal squamous cell carcinoma (ASCC). However, insufficient evidence remains to endorse microbiota modulation as a strategy to enhance tumor radiosensitivity in clinical practice. Well-designed studies focusing on prebiotics, probiotics, and fecal microbiota transplantation are needed across all GI sites to evaluate their potential to improve treatment efficacy, as suggested by promising preclinical findings. The impact of pre-treatment microbiota analyses should be addressed in prospective studies to verify the efficacy of patient-level tailored strategies. Additionally, the repurposing of radioprotective agents with innovative delivery systems, such as encapsulated amifostine, holds significant promise for mitigating small bowel toxicity, thereby enabling more effective RT treatment.

## 1. Introduction

All microorganisms symbiotically associated with the human body are collectively termed the microbiota, a complex ecosystem that plays a significant role in human health and disease. The importance of this interaction in oncology is increasingly recognized, with the microbiota now proposed to be a component of the “hallmarks of cancer” [1].

Despite this interest, defining the role of the microbiota in cancer is nonetheless challenging as its interactions with the host and neoplastic cells are highly complex and context-dependent. High-quality preclinical studies have demonstrated the microbiota’s involvement in cancer growth, suppression, therapy response, and treatment-related toxicity. However, consistency between studies is often limited, especially when translating preclinical findings into clinical contexts [2].

Recent clinical breakthroughs suggest a role for the microbiota in modulating responses to systemic therapy in solid tumors, presenting a new avenue to overcome drug resistance through microbiome modulation [3].

In several gastrointestinal (GI) cancers, radiotherapy (RT) represents a cornerstone treatment, and there is growing interest in exploring RT–microbiota interactions to improve therapeutic outcomes and reduce toxicity [4]. However, compared with systemic therapies, the specific mechanisms underlying the interaction between RT and the gut microbiota are not yet fully understood. This review provides a contemporary overview of how the microbiota influences GI epithelial homeostasis, cancer promotion, and GI cancer progression. It examines how the microbiota modulates radiosensitivity and radioresistance, and explores its role in radiotherapy-induced toxicity, with preclinical and clinical insights aiming to identify the key pathways through which the microbiota modulates the efficacy and toxicity of radiotherapy in GI tumor subsites. Finally, we discuss the implications of these findings to optimize current and future radiotherapy approaches in GI oncology.

## 2. Methods

We performed a review of the literature following PRISMA-ScR guidelines for a scoping review [5]. A literature search was performed using the MEDLINE (via PubMed) and EMBASE electronic databases. We used a search algorithm based on a combination of the following terms: microbiota OR microbioma AND radiotherapy. (The search accounted for synonyms of radiotherapy such as radiation therapy and radiation.) The search was performed until 30 June 2024. Only articles in English were selected; conference proceedings, editorials, and reviews were excluded. To expand our search, the references of the retrieved articles were also screened for additional studies. The whole review protocol is available upon explicit request. The scoping review design was chosen because of the huge heterogeneity of studies concerning the microbiota and radiotherapy, which presented different settings (preclinical, translational, and clinical), designs (retrospective and prospective), and objectives (tumor response, different toxicity outcomes, and scored at different time intervals).

### 2.1. Study Selection

Three researchers (A.B., A.G., and V.M.) independently reviewed the titles and the abstracts of the retrieved articles. Three researchers (J.B., M.K., and M.C.) then independently reviewed the full-text version of the remaining articles to determine their eligibility.

The inclusion criteria were as follows:

- Studies including esophageal cancer, liver cancer, biliary tract and pancreatic cancer, rectal cancer and anal canal cancer.
- Preclinical and clinical studies from 2010 to 2024, with an analyzed interaction between microbiota and radiotherapy concerning microbiota-modulated treatment efficacy and/or treatment toxicity.
- Studies referencing esophageal cancer, liver cancer, biliary tract and pancreatic cancer, and rectal cancer and anal canal cancer.
- Studies in English.

The exclusion criteria were as follows:

- A disease site not contemporarily treated with radiotherapy as a definitive or neoadjuvant treatment, such as stomach cancer, gallbladder cancer, colon cancer, and gastrointestinal stromal tumors (GIST).
- Rare GI cancer diseases, such as adrenocortical carcinoma, pheocromocytoma, and ampullary carcinoma.

### 2.2. Study Endpoints

**Figure 1:**
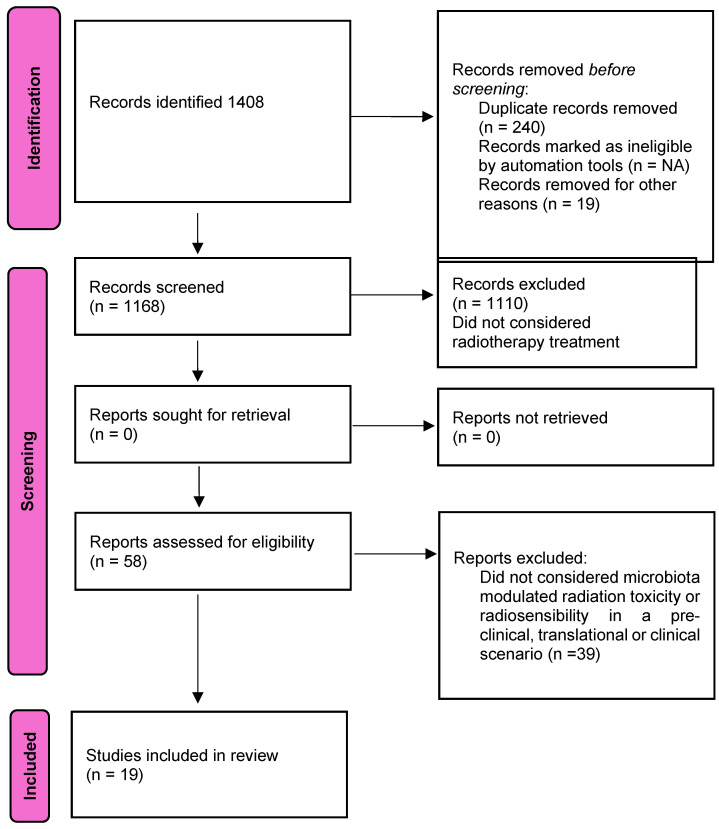
PRISMA flowchart in detail.

The study’s aim was to identify the key pathways through which the microbiota modulates the efficacy and toxicity of radiotherapy in GI tumor subsites. The PRISMA flowchart is presented in Figure 1.

## 3. Results

A total of 1408 articles were extrapolated from the first computer literature search; after applying the inclusion criteria, 1168 records remained. After the review of titles, abstracts, and full texts, 1110 records were excluded because they did not consider radiotherapy treatment. A further analysis excluded 39 studies because they did not consider microbiota-modulated radiation toxicity or radiosensibility in a preclinical, translational, or clinical scenario. A total of 19 articles were finally included in the review (Figure 1).

### 3.1. Esophageal Cancer

Exposure of a normal esophageal epithelium to both exogenous and endogenous risk factors is a well-established pathogenic mechanism in the development of esophageal squamous cell carcinoma (SCC) and adenocarcinoma (ADC). The interplay between esophageal microbiota, risk factor exposure, and tumor promotion is complex, involving multiple levels of interaction. Exposure to risk factors causes shifts in the microbiota composition to favor Gram-negative bacteria, which promote chronic inflammation and increase the potential for tumor cells to spread to lymph nodes [6,7,8].

#### 3.1.1. Microbiota Modulates Radiotherapy Efficacy in Esophageal Cancer

In a prospective study, Van Den Ende et al. analyzed fecal microbiota from 172 patients with resectable esophageal cancer undergoing concurrent chemoradiotherapy (RTCHT) [9]. Stool samples were collected at the following three critical timepoints: prior to treatment, during treatment, and post-treatment. The authors identified significant shifts in the microbial composition during and following RTCHT. By the third week of treatment, there was an observed increase in *Collinsella*, *Bacteroides*, and *Lactobacillus* populations, coupled to a decline in several *Firmicutes* taxa, including *Streptococcus*, *Ruminococcaceae*, and *CAG-352*.

Preoperative fecal samples showed elevated abundances of *Bacteroides*, *Bifidobacterium*, and *Agathobacter*, accompanied by reduced levels of *Firmicutes* species such as *CAG-352* and *Ruminococcaceae*. Notably, patients achieving a pathological complete response (pCR) exhibited higher levels of *Desulfovibrio*, *Subdoligranulum*, and *Parabacteroides*, while displaying reduced abundances of *Bifidobacterium*, *Phascolarctobacterium*, and *Escherichia coli/Shigella* compared with patients without pCR.

Progression-free survival (PFS) was positively correlated with increased levels of *Phascolarctobacterium*, *Bacteroides*, and *Bifidobacterium*, and negatively associated with the abundance of *Kosakonia*, *Romboutsia*, and *Lactobacillus*. The study concluded that greater microbiome stability throughout neoadjuvant treatment was associated with enhanced treatment responses and improved PFS outcomes.

Sasaki et al. conducted a clinical study to evaluate the composition of the gut microbiota in 51 patients with esophageal squamous cell carcinoma (SCC) undergoing active therapy. Fecal samples were collected at four distinct time points, including before treatment initiation and at subsequent intervals during and after therapy. The analysis revealed that the baseline relative abundance of *Lactobacillaceae* was higher in patients with early-stage tumors.

Focusing on microbiota dynamics during treatment and their relationship to therapeutic outcomes, the study examined 27 patients receiving concurrent chemoradiotherapy (RTCHT). Among these patients, those achieving partial or complete responses exhibited higher relative abundances of *Streptococcaceae* and *Lactobacillaceae* compared with those with progressive or stable disease. In contrast, the *Burkholderiaceae* family was more abundant in the progressive/stable disease group than in patients demonstrating favorable responses.

The authors concluded that the baseline and therapy-associated relative abundance of *Lactobacillaceae* could serve as a predictive biomarker for response to RTCHT in patients with esophageal SCC [10].

#### 3.1.2. Microbiota Modulates Radiotherapy Toxicity in Esophageal Cancer

Lin et al. conducted a study to explore the impact of microbiota modulation on treatment-related toxicity in 42 patients with esophageal cancer undergoing concurrent Radiochemotherapy (RTCHT) [11]. Fecal samples were collected at the following three time points: prior to treatment, during therapy, and at the conclusion of RTCHT. Esophageal toxicity was evaluated using the Radiation Therapy Oncology Group (RTOG) toxicity grading scale.

Among the participants, 29 patients (59.2%) experienced mild esophagitis (RTOG grades 0–1), while 20 patients (40.8%) developed severe esophagitis (RTOG grades 2–3). The latter group exhibited significantly reduced microbiota diversity and an increased relative abundance of *Fusobacterium* at the baseline compared with those without severe toxicity. Conversely, patients with mild esophagitis demonstrated a higher baseline relative abundance of *Klebsiella*, *Roseburia*, *Veillonella*, *Prevotella_9*, *Megasphaera*, and *Ruminococcus_2*.

The authors concluded that pre-treatment gut microbiota composition could serve as a predictive biomarker for the risk of severe acute radiation-induced esophagitis.

### 3.2. Rectal Cancer

The gut microbiota can promote colorectal cancer (CRC) tumorigenesis through several mechanisms. Chronic inflammation is sustained by dysbiosis, or an imbalance in microbial communities. Proinflammatory bacteria, such as *Escherichia coli* and *Enterococcus faecalis*, produce toxins and metabolites that promote DNA damage in colonic cells. Moreover, bacteria like *Bacteroides fragilis* and specific strains of *E. coli* produce genotoxins such as BFT (*B. fragilis* toxin) and colibactin. Other mechanisms are the production of carcinogenic metabolites or the downregulation of protective agents such as short-chain fatty acids (SCFAs) [12,13,14] and the evasion of immune surveillance mediated by certain bacteria, such as *Fusobacterium nucleatum*, which has been shown to bind to and inhibit natural killer (NK) cells and T cells [15,16].

These mechanisms highlight how the microbiota can create a pro-tumorigenic environment in the colon–rectum, making it a promising target for CRC prevention and treatment strategies.

#### 3.2.1. Microbiota Modulates Radiotherapy Efficacy in Colorectal Cancer

In rectal cancer, a microbiota enriched with *Bacteroides* species appears to promote tumor cell proliferation by producing purine precursors, which provide substrates essential for DNA synthesis. In a translational study, Teng et al. investigated this microbiota-mediated nucleotide synthesis pathway in a cohort of 126 locally advanced rectal cancer patients undergoing neoadjuvant chemoradiation. Stool samples were collected from patients before, during, and after treatment to observe microbiota variations over time [17].

After treatment, patients were categorized by response based on the tumor regression grade (TRG); 82 patients (TRG 0–1) were classified as responders, while 34 patients (TRG 2–3) were classified as non-responders. Initial stool analyses showed no significant baseline differences in microbiota between the two groups. A further analysis of the fecal microbiota composition revealed that *Bacteroides vulgatus* was the most enriched species in the non-responder group following chemoradiotherapy, although this enrichment was not evident before treatment. In contrast, responders had a diverse bacterial network rich in species such as *Bacteroides coprophilus*, *Rothia mucilaginosa*, and *Streptococcus thermophilus*.

The study proposed that a radioresistant phenotype in patients with a high *Bacteroides* signature could arise through the activation of nucleotide biosynthesis pathways. Specifically, this included the salvage pathways of purine and pyrimidine nucleotides as well as the salvage pathway of pyrimidine deoxyribonucleotides. A transcriptomic analysis identified the upregulation of nine genes related to purine synthesis. This upregulation was associated with higher levels of nucleotide biosynthesis substrates—hypoxanthine, uridine, guanosine, adenosine, and thymidine—in the non-responder group post-chemoradiotherapy. A colony analysis further identified *Bacteroides*, particularly *Bacteroides vulgatus*, as a primary producer of these nucleotide synthesis substrates.

In the preclinical component of this study, the underlying mechanisms of radioresistance were explored using HCT116 colorectal cancer cell lines treated with 100 mM 5-fluorouracil (5-FU) and 4 Gy radiation in the presence of a nucleoside mix. Cell survival in response to 5-FU and irradiation was assessed using MTS and colony formation assays. Supplementation with exogenous nucleosides significantly increased cancer cell survival in a dose-dependent manner under 5-FU and radiation treatment, supporting the hypothesis that nucleotide availability could enhance radioresistance.

This study provides the first translational evidence of the direct modulation of rectal cancer radiosensitivity by a microbiota profile skewed toward *Bacteroides*, particularly following treatment.

Dong et al. investigated the radiosensitizing effect of fecal microbiota transplantation (FMT) in colorectal cancer (CRC), identifying specific bacterial species that could enhance treatment response. The study analyzed 84 rectal cancer patients undergoing radiochemotherapy, categorizing them based on their response to long-course treatment and conducting stool analyses. The results showed that non-responders exhibited a reduced frequency of *Roseburia intestinalis* and *Roseburia hominis*, with *Roseburia intestinalis* being more predominant than *Roseburia hominis* and showing higher frequencies in responders. *Roseburia* species, particularly *Roseburia intestinalis*, produce butyrate, a short-chain fatty acid known for its beneficial effects on gut health.

To further assess its therapeutic role, *Roseburia intestinalis* was cultured and administered via oral gavage in mouse models of primary CRC and CRC liver metastasis. The results indicated that *Roseburia intestinalis* administration significantly improved radiosensitivity in these models.

Mechanistically, the study suggests that *Roseburia*-*intestinalis*-derived butyrate enhances radiosensitivity through the activation of the OR51E1 G-protein-coupled receptor, which is overexpressed in rectal cancer. Butyrate’s activation of OR51E1 promotes radiogenic autophagy in CRC cells, which correlates with increased RALB expression in clinical rectal cancer tissues and CRC mouse models. Inhibition of the OR51E1/RALB pathway suppressed butyrate-induced autophagy in irradiated CRC cells, further supporting its role in radiosensitization.

These findings indicate that *Roseburia intestinalis* may serve as a promising probiotic alternative to FMT to enhance radiosensitivity in CRC, potentially offering a practical therapeutic approach without the complexities of FMT [18].

Further evidence supporting the role of the gut microbiota in modulating radioresistance comes from a clinical study conducted by Sun et al. In this study, 39 patients with locally advanced rectal cancer (LARC) were enrolled. Fecal and peripheral blood samples were collected from each patient at the following three time points: prior to long-course chemoradiotherapy (CRT), during CRT (at week 3), and following CRT (at week 5). The analyses revealed distinct gut microbiome profiles and enterotype characteristics that could differentiate patients with favorable responses (AJCC TRG classification 0–1) from those with less favorable responses (TRG 2–3) to CRT.

The study introduced an integrated model that incorporated specific bacterial taxa in stool samples, blood viral profiles, and lymphocyte counts to predict patient responses to CRT. The findings showed a significant decline in bacterial diversity among patients who had a poor response to treatment. Additionally, low baseline levels of *Clostridium sensu stricto 1*, an increased fold change in the blood levels of herpesvirus entry mediator (HVEM), elevated lymphocyte counts, and a reduced fold change in *Intestinimonas* abundance from the baseline to mid-CRT were associated with a poor treatment response [19].

In a related study, Jang and colleagues collected fecal samples from 45 patients with rectal cancer prior to long-course chemoradiotherapy (CRT) to investigate microbial networks and species associated with CRT response. The analysis revealed that members of the Bacteroidales order, including the families *Bacteroidaceae* and *Rikenellaceae* as well as the genus *Bacteroides*, were more abundant in patients who did not achieve a complete response (non-CR) compared with those who achieved a complete response (CR). A metabolic pathway analysis indicated that the pathways related to anabolic functions were more prominent in CR patients. Furthermore, a Bayesian network analysis identified *Duodenibacillus massiliensis* as a key microbial species associated with an increased rate of complete response to CRT [20].

Tumor hypoxia is a known predictor of poor patient outcomes in several cancer types, partly because it reduces the efficacy of radiation therapy. In a recent study, Benej et al. analyzed RNA sequencing data from a cohort of patients treated with chemoradiotherapy (CRT) to investigate whether the modulation of tumor hypoxia could be mediated by the microbiome. A tissue analysis revealed that a high hypoxia expression score was associated with poor patient outcomes and identified tumors enriched with specific microbes, including *Fusobacterium nucleatum*. Additionally, the presence of other microbes, such as *Fusobacterium canifelinum*, was correlated with worse clinical outcomes, suggesting a potential interaction between tumor hypoxia, the microbiome, and radiation response.

To explore this interaction mechanistically, the researchers implanted CT26 colorectal cancer cells into immune-competent BALB/c mice and immune-deficient athymic nude mice. After allowing tumors to passively acquire microbes from the gastrointestinal tract, they harvested the tumors, extracted nucleic acids, and performed sequencing of both host and microbial RNA. This approach enabled the stratification of tumors based on their hypoxia score and allowed a metatranscriptomic analysis of microbial gene expression within the tumor environment. The findings indicated that tumor hypoxia gene expression scores were associated with distinct microbial populations and appeared to trigger adaptive transcriptional responses in intratumoral microbes, potentially impacting clinical outcomes [21].

#### 3.2.2. Microbiota Modulates Radiotherapy Toxicity in Colorectal Cancer

##### Enteropathy, Diarrhea, and Protective Effects of Pre- and Probiotics

Radiation enteropathy represents a dose-limiting toxicity in radiotherapy, and emerging evidence suggests that the gut microbiota plays a role in its pathogenesis. Radiotherapy-induced dysbiosis has been implicated in intestinal damage and increased susceptibility to inflammation. Research by Gerassy-Vainberg et al. demonstrated that radiotherapy can induce proinflammatory dysbiosis that promotes inflammatory susceptibility through host cytokine activation [22].

In another study, Zhang et al. investigated the protective effects of *Lactobacillus Rhamnosus* GG (LGG) on radiation-induced intestinal injury and its underlying mechanisms [23].

Mice were assigned to either a control group, a group receiving 10 Gy total abdominal irradiation (TAI), or a group pre-treated with 10^8^ CFU of LGG three days before TAI. The analysis of the small intestine and gut microbiota 3.5 days post-exposure revealed that LGG pre-treatment improved the intestinal structure and reduced DNA damage in the jejunum by inhibiting the inflammatory cGAS/STING pathway. LGG treatment also reduced the infiltration of proinflammatory M1 macrophages and CD8+ T cells and helped to restore the Th17/Treg cell balance in the inflamed jejunum. Additionally, LGG partially restored the gut microbiota composition, suggesting a therapeutic radioprotective effect through immune homeostasis and microbiota modulation.

Wang et al. explored the association between diarrhea and gut microbiota in twenty patients undergoing pelvic radiotherapy [24].

Stool samples were collected from patients and from four healthy volunteers at designated time points before, during, and after treatment. A sequencing analysis showed significant alterations in microbial diversity and richness as well as an elevated *Firmicutes*/*Bacteroidetes* ratio in patients predisposed to diarrhea before radiotherapy. Pelvic radiotherapy further altered the fecal microbial ecology.

In addition, plasma citrulline—an indicator of functional enterocyte mass—significantly decreased during radiotherapy across all patients, with more pronounced declines in those who developed diarrhea three and five weeks into treatment. Moreover, serum TNF-α levels reached significantly higher concentrations in those who developed diarrhea, particularly by the third week. These biomarkers could serve as predictive indicators for diarrhea, enabling preventive strategies to mitigate radiation-induced enteropathy.

In a randomized, double-blind, and controlled trial, Rosli et al. evaluated the efficacy of partially hydrolyzed guar gum (PHGG) as a prebiotic for preventing diarrhea in patients undergoing pelvic radiation [25].

Participants were randomly assigned to receive either 10 g PHGG or a placebo (maltodextrin) twice daily, beginning 14 days before and continuing during 14 days of pelvic radiation therapy. Diarrhea frequency, fecal microbiota composition, nutritional status, and quality of life (QoL) were assessed at the baseline and on days 14, 28 (two weeks into radiation), and 45 (two weeks after completing radiation and supplementation).

A total of thirty patients completed the trial. In the intervention group (IG), the average diarrhea frequency was initially higher than in the control group (CG) at days 14 and 28 but decreased by day forty-five. After controlling for confounders such as baseline diarrhea frequency, age, and nutritional status, a significant intervention effect was observed in diarrhea prevention at day forty-five (*p* < 0.05). Additionally, the IG demonstrated a two-fold increase in *Bifidobacterium* counts by day fourteen, a change not observed in the CG. These findings highlight the potential of prebiotics, like PHGG, to modulate gut microbiota and mitigate radiation-induced enteropathy.

Two prospective randomized trials have demonstrated the efficacy of probiotic supplementation in reducing radiation-induced enteritis among patients undergoing pelvic radiotherapy.

In a double-blind, placebo-controlled trial, Delia et al. enrolled 490 patients indicated for postoperative pelvic radiotherapy [26].

Participants were randomized to receive either a multi-strain probiotic formulation (*Lactobacillus casei*, *L. plantarum*, *L. acidophilus*, *L. Delbrueckii bulgaricus*, *Bifidobacterium longum*, *B. breve*, *B. Infantis*, and *Streptococcus thermophilus*) or a placebo. The results showed a significant reduction in radiation-induced enteritis and colitis in the experimental arm compared with the control arm (31.6% vs. 51.8%; *p* < 0.001). Additionally, severe (Grade 3–4) diarrhea occurred in only 1.4% of patients in the experimental arm compared with 55.4% in the placebo group, highlighting the protective effect of probiotics against severe gastrointestinal toxicity associated with pelvic radiotherapy.

Demers et al. conducted a randomized controlled trial to assess the efficacy of *Lactobacillus acidophilus* and *Bifidobacterium longum* supplementation (in either two or three doses) to prevent diarrhea in patients undergoing pelvic radiotherapy [27]. Patients were monitored for diarrhea severity using WHO and CTCAE criteria. A total of 229 patients, including 96 with rectal cancer, were enrolled in the study. At a 60-day follow-up, 65% of patients in the probiotic-supplemented group experienced Grade 2–4 diarrhea compared with 83% in the control group (HR 0.69; *p* = 0.04), indicating a significant reduction in moderate to severe diarrhea with probiotic intervention.

These findings collectively highlight the significance of multi-strain probiotic supplementation in preventing radiation-induced diarrhea. Based on such evidence, it is sufficient to consider multi-strain probiotic supplementation in clinical practice.

##### Fatigue

Chemoradiotherapy (CRT)-related fatigue is a debilitating symptom affecting 50–67% of patients with rectal cancer [28]. In a cross-sectional study, González-Mercado et al. investigated differences in gut microbial diversity and abundance between fifty rectal cancer patients with and without fatigue at the end of neoadjuvant CRT. Stool samples and fatigue ratings were collected, revealing that fatigued participants (n = 35) had increased abundances of *Eubacterium, Streptococcus, Adlercreutzia*, and *Actinomyces* as well as an enriched microbial sucrose degradation pathway compared with non-fatigued patients. The authors suggest that these findings provide a foundation for future trials focused on modulating gut microbiota to address CRT-related fatigue.

### 3.3. HCC

The gut microbiota can facilitate hepatocellular carcinoma (HCC) tumorigenesis through several key mechanisms, which include chronic inflammation sustained by dysbiosis, the bacterial production of metabolites and toxins such as lipopolysaccharides (LPS) from Gram-negative bacteria that activate hepatic toll-like receptors (especially TLR4), leading to inflammation, hepatocyte damage, and fibrosis, all of which create a pro-carcinogenic environment.

Bile acid metabolism and alcohol and acetaldehyde production also contribute to directly damaging hepatocytes and promoting carcinogenesis, especially in patients with non-alcoholic fatty liver disease (NAFLD) [29].

These mechanisms underline the complex interplay between the gut microbiota, inflammation, and liver health, highlighting potential therapeutic targets for preventing HCC.

#### Microbiota Modulates Radiotherapy Efficacy in Hepatocellular Carcinoma Through Immunogenic Cell Death

Li et al., in their translational study, revealed that microbiota-regulated immunogenic cell death contributes to radioresistance in HCC. The authors emphasized the complexity of the gut–liver axis, where the liver is continuously exposed to gut-derived signals—including bacterial products, food antigens, and environmental toxins—via the biliary tract, portal vein, and systemic circulation.

The study enrolled 24 HCC patients treated with external beam radiotherapy (EBRT) at either normofractionated (50–60 Gy) or moderately hypofractionated (2–3 Gy per fraction) doses. The results showed that the microbiota-derived product cyclic di-AMP, a bacterial second messenger, activates the stimulator of interferon genes (STING) pathway in dendritic cells. C-di-AMP synergizes with radiotherapy-induced tumor antigen release, particularly double-stranded DNA, to enhance dendritic cell maturation and antigen presentation, promoting IFN production and activating CD8+ cytotoxic T cells in a cGAS- and STING-dependent manner [30].

### 3.4. Anal Squamous Cell Carcinoma

In anal squamous cell carcinoma (ASCC), the microbiota influences carcinogenesis through various mechanisms, primarily by modulating inflammation, immune responses, and the tumor microenvironment. Studies highlight several key aspects, such as bacteria-induced gut inflammation; *Peptoniphilus*, *Fusobacteria*, *Porphyromonas*, and *Prevotella* have been found to be enriched in the microbiota of ASCC patients. Such a microbiota seems to enhance immune evasion and chronic inflammation [31].

#### Modulation of Radiation-Induced Toxicity Through Host Microbiota

Lin et al. performed a prospective study on ASCC patients receiving radical radiochemotherapy [32]. The authors studied the peri-tumoral microbiome and its associations with treatment-related toxicities. Anorectal swabs at the tumor site were collected before, during, and after treatment. rRNA gene sequencing was used to perform the diversity and taxonomic characterization of the microbiota. Microbial diversity and abundance were compared over time. A comparison of the microbiota profiles for the high-toxicity patient cohort versus the low-toxicity cohort was performed. High toxicity was defined as anal dermatitis G2-4 (CTCAE) or EPIC scores greater than or equal to the median.

Tumor microbial compositions significantly changed by the end of treatment, with enrichment in *Corynebacterium* at week five and *Clostridia* at the follow-up. Patients experiencing high toxicity at week 5 had higher relative counts of *Clostridia*, *Actinobacteria*, and *Clostridiales* at the baseline.

The authors concluded that radiation-induced toxicity is associated with patients’ specific microbial profiles but further studies are needed to test if microbial modulation through exogenous intervention could impact on radiation toxicity in the treatment of ASCC.

### 3.5. Pancreas

Patients with pancreatic ductal adenocarcinoma (PDAC) exhibit a radioresistant and chemoresistant phenotype. In PDAC, increased relative abundance of certain microbial taxa may influence tumor progression through various metabolites. Additionally, several studies have identified differences in intratumor microbiomes between the long-term and short-term survival of patients following treatment [33]. Among the factors contributing to treatment resistance, the microbiota appears to have a significant impact, particularly on the metabolism and absorption of chemotherapeutic drugs. Recent evidence also indicates that microbes affect the response to immunotherapy, regulate immune checkpoints, and facilitate cancer cell evasion from the immune system, thereby contributing to an unfavorable tumor microenvironment. Despite these data, no direct evidence of microbiota-modulated radioresistance exists in literature.

### 3.6. Other Strategies of Microbiota Modulation to Mitigate GI Radiation-Induced Toxicity

Radiation-induced small intestine damage is a critical dose-limiting toxicity in stereotactic body radiotherapy (SRT). SRT is a highly precise treatment modality that delivers conformal dose distributions with steep dose gradients to achieve optimal tumor targeting while minimizing normal tissue irradiation. However, the proximity of critical organs at risk (OARs) often constrains the delivery of an effective dose. This challenge is particularly pronounced in treating pancreatic and biliary tract lesions, given their anatomical proximity to the duodenum, stomach, and bowel. As a result, dose reductions—potentially compromising tumor control—are frequently necessary.

#### Modern Forms of Administration of Radioprotective Drugs to GI Sites

Amifostine is a clinically approved radioprotective agent with selective protective effects on normal tissues [34]. However, its application for intestinal radioprotection remains limited due to challenging pharmacokinetics and systemic toxicity. Zhang et al. investigated a novel oral delivery system for amifostine using the microalga *Spirulina platensis* as a microcarrier [35]. Their study evaluated the pharmacokinetics, radioprotective efficacy, and underlying mechanisms of this delivery platform. A further section of the experiment analyzed mice with an orthotopic model of colorectal cancer.

The pharmacokinetic analysis demonstrated that the amifostine-microcarrier formulation achieved improved accumulation and prolonged retention in the small intestine while reducing systemic drug concentrations in mice. Subsequent in vitro experiments irradiated enteric epithelial cells at doses of 2, 4, and 6 Gy, with and without amifostine-microcarrier treatment. The results indicated that the microcarrier-enhanced formulation significantly increased cell survival fractions following irradiation without impairing the anti-tumor effects of radiation.

The in vivo section of the study utilized an orthotopic colorectal cancer mouse model. Mice were treated with a single 12 Gy dose of irradiation, with or without an amifostine microcarrier, and sacrificed at 3 and 30 days post-treatment to assess early and subacute intestinal damage. The encapsulated amifostine group exhibited significantly improved crypt integrity in the small bowel, reduced intestinal damage, and enhanced survival rates compared with amifostine-alone or untreated groups.

A microbiota analysis revealed that amifostine treatment was associated with increased levels of beneficial bacterial families, including *Lactobacillaceae* and *Helicobacteriaceae*. Mechanistically, this preservation of microbiota diversity was linked to decreased intestinal permeability and enhanced intestinal barrier function, attributed to the production of short-chain fatty acids (SCFAs). These effects collectively contributed to maintaining small bowel integrity, study included in the review are summarized in Supplementary Table S1.

## 4. Discussion

Our review underscores the extensive body of preclinical literature exploring the modulation of tumor radiosensitivity and radiation-induced toxicity by the gut microbiota. Emerging clinical data, particularly in esophageal and colorectal cancers, support the notion that gut microbiota composition plays a significant role in influencing treatment toxicity.

In terms of treatment efficacy, observational studies in rectal cancer suggest that microbiota richness, particularly in *Roseburia intestinalis*, and relative abundance of species such as *Bacteroides coprophilus*, *Rothia mucilaginosa*, and *Streptococcus thermophilus*, are associated with favorable treatment responses, indicating a radiosensitizing effect. Conversely, a relative enrichment of certain *Bacteroides* species has been linked to tumor radioresistance, mediated by the production of purine precursors. Similarly, in hepatocellular carcinoma (HCC), bacterial c-di-AMP has been shown to stimulate the immune system via the STING pathway, with the restoration of gut homeostasis in dysbiotic conditions promoting immunogenic cell death. However, these findings are largely confined to normofractionated and moderately fractionated external beam radiotherapy (EBRT). Nevertheless, the identification of a key microbiota signature identifying patients more at risk of radiotherapy-induced toxicity may support the use of microbiota analyses ab initio to select patient candidates for tailored support strategies.

A notable gap in the literature concerns the applicability of these findings to modern stereotactic body radiotherapy (SBRT) regimens. SBRT delivers higher doses per fraction over shorter durations (3–8 days), potentially increasing antigen release but raising questions about the relevance of microbiota-mediated immunogenic cell death in this context. Future studies are needed to clarify the microbiota’s role in enhancing SBRT efficacy and to investigate its impact in such high-dose, short-course treatment settings.

Another significant limitation of existing studies is their observational design, which identifies the microbiota profiles in good and poor responders but fails to establish causal relationships or evaluate the potential for the exogenous modulation of the microbiota to improve outcomes. Although no randomized evidence currently supports microbiota modulation to enhance tumor radiosensitivity, the bacterial signatures identified in observational studies provide a foundation for future interventional trials.

In contrast, prospective translational studies support the exogenous modulation of the gut microbiota to reduce radiation-induced toxicity. Effective interventions include prebiotic supplementation, such as partially hydrolyzed guar gum (PHGG), and prophylactic multi-strain probiotic administration. These strategies are particularly relevant given the logistical and clinical complexities of fecal microbiota transplantation (FMT). Although preclinical data on FMT are promising, clinical evidence remains sparse and FMT faces challenges related to treatment reproducibility, donor selection, and safety concerns. This is reflected in the present review, where a lack of studies concerning FMT and RT is evident, probably due to challenges in the use of FMT in clinical scenarios [36]. Consequently, the current focus has shifted toward prebiotics and probiotics, which offer more consistent and manageable approaches to microbiota modulation.

Additionally, the repurposing of amifostine using innovative delivery systems, such as microcarrier platforms, represents a novel avenue to protect the gut from radiation-induced damage. Beyond its direct radioprotective effects, preclinical evidence suggests that amifostine may indirectly preserve gut microbiota diversity, contributing to improved intestinal barrier function and reduced toxicity. These findings are particularly promising for upper gastrointestinal (GI) targets, such as pancreatic and biliary tract cancers, where the radiosensitivity of adjacent organs (e.g., the duodenum, stomach, and small bowel) presents significant challenges in delivering effective therapeutic doses. Combining advanced radiotherapy techniques, optimized contouring and planning strategies, and innovative radioprotective modalities may enhance treatment safety and efficacy for these challenging clinical scenarios [37,38,39,40,41,42,43,44,45].

Despite the growing enthusiasm for leveraging microbiota modulation to improve oncologic outcomes, this review highlights the inherent challenges in translating robust preclinical evidence into clinical practice. Notably, the findings from preclinical models are not always replicated in clinical studies. This discrepancy can be attributed, in part, to the intricate nature of the human gut microbiota and the multifaceted interactions between bacterial species, microbial metabolites, host tissue cells, and tumor cells. Such complexity underscores the need for a careful study design when attempting to validate preclinical observations in human clinical scenarios.

Our study presents some important limitations. A lack of consistency for both toxicity and efficacy endpoints between studies limits the possibility to perform a systematic review and to provide a measure of effect.

This aspect is critical and not easily manageable given the enormous microbiota environment and the multifaced level of cross-talk between each bacterial or microbiomal product and normal and cancer cells. Moreover, given the differences in study designs and their frequent observational/retrospective nature, a rigorous estimation of the risk of bias for each study included following contemporary guidelines is lacking.

Future translational research must account for the dynamic and individualized nature of the microbiota as well as its context-dependent effects on health and disease. Rigorous methodologies, including longitudinal sampling, comprehensive microbial profiling, and consideration of host factors such as diet, medication, and immune status, are essential. Only through such a meticulous study design can we effectively bridge the gap between promising in vitro or animal model findings and tangible clinical outcomes.

## 5. Conclusions

The impact of the microbiota on radiation-induced toxicity and cancer response to radiotherapy is an emerging area of interest. Multiple studies support the clinical application of microbiota modulation, particularly through prebiotics and probiotics, to prevent or mitigate radiotherapy-induced toxicity in rectal cancer. Some evidence also suggests that, in esophageal cancer and ASCC, microbiota modulation may play a role in the management of RT toxicity. Concerning the modulation of efficacy, despite the interesting results of the studies of rectal cancer, esophageal cancer, and HCC, the evidence remains insufficient to endorse microbiota modulation as a strategy to enhance tumor radiosensitivity in clinical practice. Well-designed studies focusing on prebiotics, probiotics, and fecal microbiota transplantation are essential across all GI sites to evaluate their potential to improve treatment efficacy, as suggested by promising preclinical findings. The impact of pre-treatment microbiota analyses should be addressed in prospective studies to verify the efficacy of patient-level tailored strategies. Additionally, the repurposing of radioprotective agents with innovative delivery systems holds significant potential for mitigating small bowel toxicity through microbiota preservation, thereby enabling more effective RT treatment.

## Supplementary Materials

The following supporting information can be downloaded at: https://www.mdpi.com/article/10.3390/cimb47040265/s1.
