# Supplementary material for: Microbiota Modulation of Radiosensitiveness and Toxicity in Gastrointestinal Cancers: What Radiation Oncologists Need to Know—A Review on Behalf of the Italian Association of Radiobiology (AIRB)
Source: Curr Issues Mol Biol. 2025 Apr 9;47(4):265. doi: 10.3390/cimb47040265 (PMC12025633; doi:10.3390/cimb47040265)
Supplement: Supplementary file 1 [file cimb-47-00265-s001.zip › cimb-3511468-supplementary.pdf]

GI SUBSITE	STUDY	TOPIC: MODULATION OF RT TOXICITY/EFFICACY	STUDY CONTEXT	MAIN STUDY RESULTS
ESOPHAGUS	Van Den Ende et al 2024	EFFICACY: RTCHT and surgery for 172 pts with esophageal cancer	Clinical	Patients achieving pCR: higher levels of *Desulfovibrio*, *Subdoligranulum*, and *Parabacteroides*, while displaying reduced abundances of *Bifidobacterium*, *Phascolarctobacterium*, and *Escherichia coli/Shigella* compared to patients without pCR.
	Sasaki et al. 2023	EFFICACY: radical therapy for 51 pts with esophageal SCC	Clinical	Patients achieving partial or complete responses exhibited higher relative abundances of *Streptococcaceae* and *Lactobacillaceae* compared to those with progressive or stable disease. In contrast, the *Burkholderiaceae* family was more abundant in the progressive/stable disease group
	Lin et al. 2022	TOXICITY: 42 patients with esophageal cancer in RTCHT	Clinical	Patients with mild esophagitis demonstrated a higher baseline relative abundance of *Klebsiella*, *Roseburia*, *Veillonella*, *Prevotella_9*, *Megasphaera*, and *Ruminococcus_2*.
RECTUM	Teng et al 2023	EFFICACY: 126 patients undergoing neoadjuvant RTCHT	Translational	*Bacteroides vulgatus* was the most enriched species in the non-responder group following chemoradiotherapy, In contrast, responders had a diverse bacterial network rich in species such as *Bacteroides coprophilus*, *Rothia mucilaginosa*, and *Streptococcus thermophilus*.
	Dong et al. 2024	EFFICACY: 84 patients undergoing RTCHT	Translational	*Roseburia intestinalis* may serve as a promising probiotic alternative to FMT for enhancing radiosensitivity.
	Sun et al. 2023	EFFICACY: 39 patients undergoing neoadjuvant RTCHT	Clinical	Significant decline in bacterial diversity among patients who had a poor response to treatment. Additionally, low baseline levels of *Clostridium sensu stricto 1*, an increased fold change in blood levels of the herpesvirus entry mediator (HVEM), elevated lymphocyte counts, and a reduced fold change in *Intestinimonas* abundance from baseline to mid-CRT all related with poor treatment response
	Jang et al. 2020	EFFICACY: 45 patients undergoing neoadjuvant RTCHT	Clinical	Members of the Bacteroidales order, including the families *Bacteroidaceae* and *Rikenellaceae*, as well as the genus *Bacteroides*, were more abundant in patients who did not achieve complete response. *Duodenibacillus massiliensis* as a key microbial species associated with an increased rate of complete response to CRT
	Benej et al. 2024	EFFICACY human tumor tissue analysis + CT26 colorectal cancer cells in mice	Pre-clinical	Tissue analysis revealed that a high hypoxia expression score was associated with poor patient outcomes and identified tumors enriched with specific microbes, including *Fusobacterium nucleatum*. Pre-clinical model findings indicated that tumor hypoxia gene expression scores were associated with distinct microbial populations and appeared to trigger adaptive transcriptional responses in intratumoral microbes.
	Zhang et al. 2024	TOXICITY Mice 10 Gy total abdominal	Pre-clinical	possible therapeutic radioprotective effect of probiotics *Lactobacillus rhamnosus* in alleviating RT

		irradiation		induced intestinal injury by maintaining immune homeostasis and reshaping gut microbiota.
	Wang et al. 2015	TOXICITY 20 patients undergoing pelvic RT	Clinical	Elevated Firmicutes/Bacteroidetes ratio in patients predisposed to diarrhea before radiotherapy.
	Rosli et al. 2020	TOXICITY 30 RTCHT pts	Clinical; RCT	In experimental arm, partially hydrolyzed guar gum (PHGG) potentially increased the *bifidobacterial* count and seemed to have post-supplementation effects by reducing the frequency of diarrhea upon treatment completion.
	Delia et al. 2007	TOXICITY; 490 pelvic RT pts	Clinical; RCT	Significant reduction in RT-induced enteritis and colitis in the experimental arm (supplement with: *Lactobacillus casei, L. plantarum, L. acidophilus, L. Delbrueckii bulgaricus, Bifidobacterium longum, B. breve, B. Infantis,* and *Streptococcus thermophilus*) compared to the control arm.
	Demers et al. 2013	TOXICITY 229 pelvic RT pts	Clinical; RCT	Probiotic supplementation (*Lactobacillus acidophilus* and *Bifidobacterium longum*) reduced RT induced diarrhea at the end of the treatment in experimental arm.
	González-Mercado et al. 2020	TOXICITY 50 patients undergoing neoadjuvant RTCHT	Clinical	Fatigued patients had increased abundances of *Eubacterium, Streptococcus, Adlercreutzia*, and *Actinomyces*, as well as an enriched microbial sucrose degradation pathway, compared to non-fatigued patients.
LIVER	Li et al. 2022	EFFICACY 24 HCC pts (RT) at either normofractionated (50–60 Gy) or moderately hypofractionated	Translational	Microbiota-regulated immunogenic cell death contributes to radioresistance in HCC via C-di-AMP /stimulator of interferon genes (STING) pathway
ANAL CANAL	Lin et al. 2022	TOXICITY Radical RTCHT	Clinical	Patients experiencing high toxicity at week 5 had higher relative counts of *Clostridia*, *Actinobacteria*, and *Clostridiales* at baseline.
SMALL BOWEL§	Zhang et al. 2022	TOXICITY Mice, small bowell irradiation	Pre-clinical	Encapsulated Amifostine with spirulina microcarrier significantly improved crypt integrity in the small bowel, reduced intestinal damage, and enhanced survival rates compared to Amifostine-alone or untreated groups.Moreover, increased levels of beneficial bacterial families, including Lactobacillaceae and Helicobacteriaceae was observed in the treatment group

LEGENDA: RTCHT= radiochemotherapy; pCR= pathologic complete response; SCC= squamous cell carcinoma; FMT= fecal microbiota transplant; RT= radiotherapy

§ CONSIDERED AS ORGAN AT RISK TO PROTECT
